# Gap junction structure: unraveled, but not fully revealed

**DOI:** 10.12688/f1000research.10490.1

**Published:** 2017-04-26

**Authors:** Eric C. Beyer, Viviana M. Berthoud

**Affiliations:** 1 Department of Pediatrics, University of Chicago, Chicago, IL, 60637, USA

**Keywords:** gap junction channel, connexin26, crystal structure, membrane topology, intercellular communication

## Abstract

Gap junction channels facilitate the intercellular exchange of ions and small molecules, a process that is critical for the function of many different kinds of cells and tissues. Recent crystal structures of channels formed by one connexin isoform (connexin26) have been determined, and they have been subjected to molecular modeling. These studies have provided high-resolution models to gain insights into the mechanisms of channel conductance, molecular permeability, and gating. The models share similarities, but there are some differences in the conclusions reached by these studies. Many unanswered questions remain to allow an atomic-level understanding of intercellular communication mediated by connexin26. Because some domains of the connexin polypeptides are highly conserved (like the transmembrane regions), it is likely that some features of the connexin26 structure will apply to other members of the family of gap junction proteins. However, determination of high-resolution structures and modeling of other connexin channels will be required to account for the diverse biophysical properties and regulation conferred by the differences in their sequences.

## Introduction

Intercellular communication through gap junction channels is critical for integrating the functions of cells in the tissues of all multicellular organisms by allowing direct exchange of ions and small molecules. In vertebrates, gap junctions are formed by members of a family of proteins that are called connexins (Cx). The importance of gap junctions is supported by the wide range of abnormalities linked to connexin mutations or caused by disrupting connexin expression (including deafness, neuropathies, arrhythmias, cataracts, skin diseases, lymphedema, and impaired development) (reviewed in 1– 3). Understanding the structures of the channels formed by connexins is critical for elucidating gap junction channel permeability and regulation at an atomic level.

**Figure 1.**
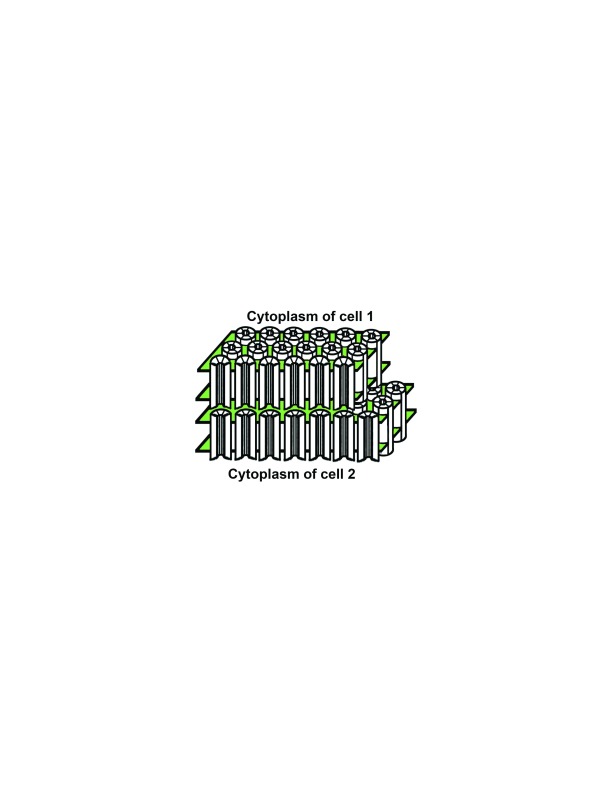
Diagram of the gap junction structure. A gap junction is a cluster of intercellular channels formed by head-to-head apposition of connexin hemichannels within the plasma membranes of adjacent cells. The hemichannels are hexameric assemblies of connexin proteins surrounding a central aqueous pore and are depicted as cylinders formed of six subunits. The boundaries of the plasma membrane are illustrated in green. The model is based on Makowski *et al*. ^8^.

Gap junctions were originally defined based on structure (appearance in electron micrographs) ^4– 6^. They also were among the first membrane channels for which low-resolution three-dimensional structural analyses were accomplished. Nearly 40 years ago, isolated liver gap junctions were subjected to electron microscopy and X-ray diffraction crystallography followed by image analysis ^7, 8^. These studies led to a model in which the gap junction channel is formed of two hemichannels (one contributed by each of the apposing cells) that dock to each other (“head-to-head”); each hemichannel (also called a connexon) contains six subunit proteins (connexins) that surround the central water-filled pore ( Figure 1). Over subsequent years, this model was refined by additional structural studies of two-dimensional arrays of channels in isolated gap junctions ^9, 10^.

**Figure 2.**
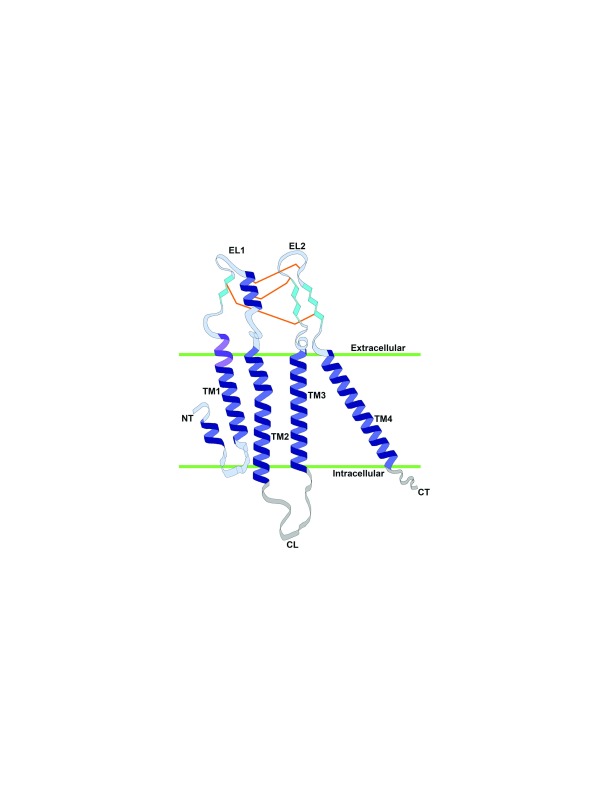
Two-dimensional representation of the structure of a connexin26 (Cx26) monomer within the membrane. The structure of Cx26 is indicated based on Maeda *et al*. ^21^. Protein secondary structure is colored as follows: deep blue, α-helix; purple, parahelix; turquoise, β-sheet. Amino acids whose side chains are included in the Cx26 structure but are not part of a helix or β-sheet are colored light blue; disordered regions are depicted in gray pattern. Disulfide bonds formed between cysteine residues are indicated by orange lines. The boundaries of the plasma membrane (green lines) delimiting the extracellular and intracellular sides are based on Kwon *et al*. ^23^. Domains within the connexin monomer are also indicated: CL, cytoplasmic loop; CT, C-terminus; EL1 and EL2, extracellular loops 1 and 2; NT, N-terminus; TM1–TM4, transmembrane domains 1–4.

Cloning of DNA sequences and predictions regarding the encoded polypeptides suggested that all of the connexins had similar membrane topologies and each contained four transmembrane domains (TMs) ^11– 14^. In the connexin membrane topology, the N-terminus (NT), a cytoplasmic loop (CL) between TM2 and TM3, and the C-terminus (CT) are cytoplasmic, while two domains, EL1 (between TM1 and TM2) and EL2 (between TM3 and TM4), are extracellular ( Figure 2). This connexin topology model was supported by immunoelectron microscopy studies mapping different peptide sequences in the connexin molecule ^15, 16^. A large number of functional studies involving site-directed mutagenesis and physiological characterization of the properties of expressed mutant connexins were performed based on these models. Electron cryo-crystallography allowed determination of the structure of channels formed by a truncated connexin43 (Cx43), lacking most of the CT, at 7.5 Å resolution; it showed the presence of 24 transmembrane helices in each hemichannel, consistent with the initial models ^17^. NMR analysis of synthetic peptides showed the presence of short α-helices within the N-terminal domains of several connexins ^18– 20^.

## Connexin26 crystal structure

The understanding of gap junction structures was significantly advanced in 2009, when Maeda and colleagues published a 3.5 Å X-ray crystal structure of the human connexin26 (Cx26) channel ^21^. This structure confirmed many aspects of the existing gap junction channel models, including the dodecameric channel structure (formed of two hexameric hemichannels) and the presence of four α-helical TMs in each connexin monomer. The Cx26 structure showed that the permeation pathway through the hemichannel contained a positively charged cytoplasmic entrance, a funnel, a negatively charged path, and an extracellular cavity. The structure resolved a previous controversy by showing that the major pore-lining helix was TM1. However, the structure did not allow resolution of the positions of the side chains of some critical N-terminal amino acids. The CL (residues 110–124) and the short cytoplasmic carboxyl terminal segment (218–226) could not be modeled because of a lack of order in these regions. Many features of the Cx26 gap junction channel have been confirmed by an independent crystal structure ^22^.

The Cx26 structure has provided a basic atomic model to investigate the function and regulation of Cx26 channels in more detail. In addition, because high-resolution structures of other members of the connexin family have not yet been determined, it has been utilized to predict features of other connexin channels and the consequences of their mutants.

## Structural insights into channel permeability and conductance

Availability of the Cx26 structure has driven further investigations of gap junction channel permeability. Because this structure contains an unobstructed permeation pathway with a pore diameter of ~14 Å (based on minimal center-to-center distances of opposed heavy atoms and without considering atom diameters) and because crystallization was performed at neutral pH and in the absence of divalent ions, Maeda *et al*. suggested that it was an open channel ^21^. However, Kwon *et al*. ^23^ pointed out that Maeda *et al*. ^21^ may have overestimated the pore diameter because their Cx26 structure does not contain coordinates of the N-terminal methionine (which is acetylated as determined by mass spectrometry ^24^) or of the side chains of several N-terminal domain amino acids that may contribute to restricting the aqueous pore. When they accounted for these additional features, Kwon *et al*. estimated that the pore of the published Cx26 structure would actually be <6 Å, a size too small to allow the passage of hydrated ions or the molecular tracers that permeate through gap junction channels ^23^. To generate a structure that may more accurately represent the “open” configuration, Kwon *et al*. used molecular dynamics simulations to equilibrate the Cx26 hemichannel after completion of the structure by the addition of the missing residues; then, they utilized Brownian dynamics to test the validity of the equilibrated model by comparing the current-voltage relation of the refined atomic structure calculated with grand canonical Monte Carlo Brownian dynamics with that of the experimental data ^23^. This equilibrated structure has an average pore diameter of 12.8 Å (at the position of M1). Similar pore diameters were obtained by Zonta *et al*. for human Cx26 and Cx30 using molecular dynamics ^25^.

The cytoplasmic channel entrance is formed by parts of TM2 and TM3, where a concentration of positively charged amino acid side chains may facilitate the accumulation of negatively charged permeant molecules ^26^. If this aspect of the Cx26 structure was a major determinant of channel permeability for all connexin channels, they should all preferentially permeate negatively charged molecules. However, the K/Cl permeability ratio for intercellular Cx26 channels is about 2.6 ^27^. In addition, several other connexins have greater permeabilities for positively than for negatively charged ions and larger molecules ^28– 30^. In their modeling, Kwon *et al*. concluded that co- or post-translational modification (acetylation) of the N-terminal methionine and of several lysine residues at the cytoplasmic pore entrance may greatly influence charge selectivity ^23^.

The pore funnel is formed by six NT helices that gradually narrow the pore diameter ^21^. The charges of amino acid side chains in the NT region are determinants of the unitary conductance and charge selectivity of connexin channels, as demonstrated by mutagenesis studies creating substitutions or deletions in the NT that produced alterations of these properties ^31– 37^. Zonta *et al*. modeled Cx30 channels and concluded that differences in unitary conductance between Cx26 and Cx30 might result from differences in positions and charges of ion sieves within their pores ^25^. Modeling based on the Cx26 structure has been used to develop atomic level interpretations of the results from studies of disease-related NT mutants of other connexins like Cx46 ^38, 39^.

Additional negative charges along the permeation pathway (contributed by side chains of pore-lining residues, especially at the TM1/E1 boundary) may also contribute to channel properties ^40– 45^. This region may play a major role in determining the permeability of Cx26 channels. It is possible that a positively charged permeant undergoes sequential, transient interactions with negatively charged side chains as it progresses through the pore until it reaches the cytoplasmic entrance of the connexin channel in the adjacent cell. Computational methods to simulate the movement of a permeant and an impermeant sugar molecule through the Cx26 gap junction channel led to the conclusion that their selective permeation may be influenced by hydrogen bonding, interaction with K ^+^, and “entropic factors arising from permeant flexibility” ^46^.

Molecular dynamics simulations of the human Cx26 hemichannel identified a water pocket (also termed intracellular or IC pocket) located between the four TM domains of each connexin monomer, totaling six water pockets per hemichannel ^47^. The water in these pockets may be able to move between the pocket and the main channel via a small aperture below the NT domain; however, the diffusion coefficient of water molecules in the pocket varies depending on the connexin monomer, and the residence of the water molecules is different in each pocket. The water pocket has been hypothesized to have a role in hemichannel activity (including permeability) ^47^.

A tryptophan-scanning mutagenesis study revealed extensive interactions between TMs in Cx32 that are critical for gap junction function ^48^. This study suggested that the Cx32 gap junction channel has a similar architecture of TMs to both the Cx26 structure (on which it was based) and its equilibrated molecular dynamics model, including the presence of IC pockets ^48^.

## Docking of hemichannels

Gap junction channels are unique when compared to other channels in that they span the plasma membranes of two adjacent cells and allow intercellular communication. The formation of the gap junction channel requires docking between two hemichannels and the production of a tight seal to separate the channel interior from the extracellular milieu. Given the membrane topology of connexins and that the channel spans two plasma membranes, docking must involve the extracellular domains.

Several studies have contributed to elucidating the structure of the gap junction channel in the extracellular gap. Cysteine scanning mutagenesis suggested that the extracellular domains contain anti-parallel β-sheets and that the ELs from one hemichannel interdigitate with those of the opposing hemichannel, forming a β-barrel extension of the gap junction channel at the docking interface ^49^. Cryo-electron microscopy of a truncated Cx43 channel showed that the extracellular region was double layered, including a continuous inner layer of protein capable of forming a tight seal ^17^. Specific EL1-EL1 and EL2-EL2 intercellular interactions have been identified in the Cx26 crystal structure ^21^. In EL1, N54 forms hydrogen bonds with the main-chain amide of L56 from the opposite connexin in the opposed hemichannel, and Q57 forms hydrogen bonds with Q57 of the diagonally opposite connexin in the opposed hemichannel. In EL2, K168, D179, and the main-chain carbonyl groups of T177 and N176 form hydrogen bonds and salt bridges with the opposite connexin in the opposed hemichannel. Together, these interactions and those between the connexin monomers forming each hemichannel result in the formation of a tight, double-layered wall spanning the extracellular gap, connecting the opposed hemichannels.

The docking of other connexins may involve similar interactions to those identified in Cx26. Among the human connexins, the amino acid corresponding to Q57 is absolutely conserved, and those corresponding to N54 and D179 are well conserved (differing in only two or three of the 20 family members). However, amino acids corresponding to L56, K168, N176, and T177 diverge in many of the connexins. To ensure the formation of a tight seal between apposed hemichannels formed by other members of the family, there may be compensatory substitutions of the corresponding amino acid residues to restore hydrogen bond formation with the differing interacting amino acids, or, alternatively, there may be compensatory interactions between surrounding residues that differ from those of Cx26.

Knowledge of the amino acid residues important for docking has helped elucidate the molecular mechanism of malfunction of some disease-associated connexins. In their studies of a cataract-linked mutant (Cx46N188T), which forms functional hemichannels (but not intercellular channels), Schadzek *et al*. constructed models of Cx46 based on the Cx26 structure that allowed them to conclude that the mutated amino acid is critical for the docking of hemichannels through the formation of hydrogen bonds with the opposing hemichannel ^50^. The mutated amino acid in Cx46 (N188) corresponds to N176 of Cx26. A mutant of Cx32 at the corresponding position (Cx32N175D) causes X-linked Charcot-Marie-Tooth disease and, similarly, does not form functional homotypic gap junction channels ^51^.

Pairing of hemichannels containing different connexins requires specificity of connexin interactions to form functional heterotypic intercellular channels. This process has been studied by bringing cells (or *Xenopus* oocytes) expressing one connexin subtype into apposition with cells (or *Xenopus* oocytes) expressing a different connexin subtype and assessing gap junctional conductance. Some combinations of connexins result in functional channels (i.e. compatible connexins) and some do not (i.e. incompatible connexins). For example, Cx26 forms heterotypic channels with Cx32, but not with Cx40 or Cx43 (see a more comprehensive diagram summarizing compatible and incompatible connexins in 52). Amino acids in EL2 have been implicated in determining connexin compatibility for the formation of functional heterotypic channels, based on the results of expression studies of chimeric connexins in which the EL1 or EL2 domains were replaced with those from a different, incompatible connexin ^53, 54^. Homology modeling and studies of the ability of Cx32 mutants to form heterotypic channels with Cx26 suggest that at least four hydrogen bonds between a pair of opposed EL2 domains are required for proper docking and functional heterotypic channel formation ^51^.

## Structural insights into channel gating

The opening and closing of connexin channels (and hemichannels) can be modulated by voltage, pH, divalent cations, a variety of chemicals, and co-/post-translational modifications. Connexin channels exhibit two functionally distinguishable voltage-dependent gating mechanisms: (1) V _j_ or fast gating, in which the channel closes to a substate as determined by a single connexin subunit ^55^, and (2) “loop” or slow gating, in which a cooperative, concerted process fully closes the channel ^56^. For both voltage-sensitive mechanisms, the voltage sensed is that within the pore and, because of this, there is interaction between the voltage-sensitive gating mechanisms. Snipas *et al*. recently analyzed this quantitatively and generated a stochastic 36-state model of gap junction channel gating ^57^. The variety of factors that modulate connexin channel function suggests that the amino acids involved in sensing them are different. Once a factor is sensed, a response must be transduced, likely through an initial conformational change. Since the sensing amino acids differ, the initial conformational change would be expected to be relatively specific. However, it is unclear whether the initial changes are followed by conformational changes that are specific for different factors or whether the changes triggered by different factors share some similarities before converging upon a common mechanism that gates the channel closed. These issues have been illustrated by studying the regulation of mutant Cx26 hemichannels. Wild-type Cx26 hemichannel activity is inhibited by both Ca ^2+^ and acidification; however, while Ca ^2+^ regulation is retained by Cx26N14K and Cx26A40V hemichannels (although modestly impaired in Cx26A40V), pH regulation is absent in Cx26N14K and severely impaired in Cx26A40V ^58, 59^. Thus, although both Ca ^2+^ and pH act on the loop gate, they promote different conformational pathways leading to closure of this gate ^59^.

Electron crystallographic analysis of a Cx26 mutant (Cx26M34A) showed a “plug” within the mouth of the channel; this density was decreased in a Cx26 mutant with a deletion of several amino acids within the N-terminal region, suggesting that connexin channels might be closed by a “plug gating” mechanism in which the NTs physically block the channel pore ^60^. Because a large body of data has shown that amino acids within the NT serve as a voltage sensor and determine the polarity of V _j_ (or fast) gating ^55, 61, 62^, it is tempting to propose that this “plug gating” mechanism could explain this voltage-dependent gating process. However, “plug gating” may not explain V _j_ (or fast) gating because Cx26M34A hemichannels exhibit a low level of conductance (when paired with wild-type Cx26 hemichannels) and show normal V _j_ gating in this asymmetric configuration ^60^. Moreover, in the tryptophan-scanning study, it was suggested that gating occurs as a result of global conformational changes around TM1 ^48^.

Loop (or slow) gating of connexin hemichannels and intercellular channels depends on amino acids at the TM1/EL1 interface ^63, 64^. Based on molecular dynamics simulations, Kwon *et al*. suggested that the 3 _10_ helix located in this region (which they termed the “parahelix” because it does not strictly fit the structural requirements of a 3 _10_ helix) contains charged residues that might act as a voltage sensor and that concerted movements of this region and other parts of the molecule might close the hemichannel ^56^. Interactions involving the NT may also be involved in loop gating, as implicated by studies of deafness-associated mutations of Cx26 involving substitutions of N14 ^59^.

Gap junction channels can also be closed by acidification. For some connexins, like Cx43 (but not others, like Cx45), acidification-induced closure of the channel may occur through a particle-receptor mechanism in which the C-terminal domain binds to residues within the CL ^65– 68^. Unfortunately, the structural basis for this form of channel closure is not well understood, since high-resolution X-ray and electron microscopy structures are not available for these connexins. NMR has been used to study the solution structures of the C-terminal domains of Cx43 (full-length or shorter peptides) and have identified α-helical regions connected by more flexible loops that may change conformation in response to pH ^69^. Interestingly, a direct interaction between the CL and the CT of Cx26 is necessary to keep the channels open; its destabilization by protonated aminosulfonates leads to channel closure ^70^.

To gain insights into gating by divalent cations, Bennett *et al*. determined the X-ray structures of the human Cx26 gap junction channel crystallized in the presence or absence of calcium ions ^22^. These two structures showed rather small conformational differences but identified calcium coordination sites (the carboxylate of E47 and carbonyl oxygen of G45 from one monomer and the carboxylate of E42 in the adjacent monomer). These amino acids are located near the beginning of the extracellular part of the channel. An earlier study had shown ɤ-carboxylation of E42 and E47 of Cx26 by mass spectrometry and suggested that this modification generated high-affinity Ca ^2+^-binding sites ^24^. Quantum chemistry computations performed by Zonta *et al*. had also suggested that Ca ^2+^ binds to the ɤ-carboxylated E47, altering the local structure of the Cx26 hemichannel and preventing stabilizing interactions with R75 from the same monomer and R184 from an adjacent monomer ^71^. These studies differ in the proposed mechanisms of Ca ^2+^-dependent block: Bennett *et al*. suggested that binding of calcium ions generates an electrostatic barrier that inhibits the permeation of cations through the pore ^22^; in contrast, Zonta *et al*. proposed that extracellular Ca ^2+^ binding produces structural alterations ^71^. It is unclear whether the Ca ^2+^ electrostatic barrier mechanism (which does not produce steric occlusion of the pore) or the structural alterations (obtained by quantum chemistry computations) could explain loop gating.

While the crystallization analysis of Ca ^2+^ interactions with Cx26 considered dodecameric intercellular channels ^22^, many of the functional studies of Ca ^2+^ block used physiological and biophysical approaches to study connexin hemichannels. Blockade of undocked connexin hemichannels has obvious biological importance as a mechanism to prevent cytotoxicity; opening of unopposed hemichannels can be prevented by their exposure to the substantial concentrations of Ca ^2+^ normally present extracellularly. Although the Ca ^2+^ block of intercellular channels and the block of hemichannels both involve amino acid residues in the ELs, these processes may not occur through identical mechanisms because docking could alter the spatial orientations of some residues and their side chains, affecting the Ca ^2+^ binding sites.

Physiological dissection of the mechanisms of Ca ^2+^-dependent block of Cx26 channels was stimulated by the discovery that some patients with keratitis-ichthyosis-deafness disorder have mutations (especially substitutions of D50) that cause aberrant opening of hemichannels due to Ca ^2+^ insensitivity ^43, 72, 73^. Expression studies suggest that Ca ^2+^ stabilizes the closed state of wild-type Cx26 hemichannels, and a negative charge at amino acid 50 is required for this stabilization ^43, 73^. Some (but not all) data suggest that D50 forms a salt bridge with the nearby K61 (from an adjacent connexin subunit) that stabilizes the open state of the Cx26 hemichannel in low external Ca ^2+^, an interaction that is disrupted in high extracellular Ca ^2+^ ^43, 73^. Although data supporting a D50-Q48 interaction have also been reported, this interaction would not make a major contribution to stabilizing the open state in low or absent external Ca ^2+^ ^74^. Lopez *et al*. used molecular dynamics simulations to identify Ca ^2+^ interaction sites and their effects on the Cx26 hemichannel ^75^. Their data suggest that E47 and D50 are part of the Ca ^2+^ binding site and that there is an electrostatic network near the extracellular entrance of the pore (including the D50-K61 salt bridge and interactions involving D46, E47, R75, R184, and E187) ^75^. Their modeling was supported by mutagenesis of critical residues in Cx26 and of the corresponding residues in Cx46 (suggesting generalizability). Furthermore, Lopez *et al*. concluded that the Ca ^2+^-sensing ring is distinct from the gate and that the gate localizes further into the pore (below G45) ^75^. Earlier studies of Cx46 hemichannels indicate that its Ca ^2+^ gate is extracellular to L35 (corresponding to M34 in Cx26) ^76^. The results from these studies would suggest that the Ca ^2+^-responsive gate in Cx26 localizes between M34 and G45.

Other regions in other connexin isoforms may also be responsible for or contribute to channel gating by divalent cations. Studying Cx32 hemichannels, Gómez-Hernández *et al*. concluded that Ca ^2+^ bound to D169 and D178 in EL2, blocking both voltage-gated opening to the higher conductance open state (90 pS) and ion conduction through partially open (18 pS) hemichannels ^77^. The charge at the amino acid corresponding to D178 is conserved in 18 of the 20 family members, but that of D169 is not. Whether this is a common mechanism for Ca ^2+^-dependent regulation of a subset of connexins as suggested by the authors remains to be evaluated. Other physiological studies have suggested that Ca ^2+^ itself or Ca ^2+^/calmodulin bind near the cytoplasmic face of the channel to gate channels closed ^78– 81^. Ca ^2+^/calmodulin binding sites have been identified for several different connexins within their NT, CL, or CT domains, and the α-helical content of CL or CT peptides containing the putative calmodulin binding sites increases after binding calmodulin ^82^. Because both the CL and the CT also possess unstructured regions, it is possible that calmodulin binding leads to movements of these domains that may obstruct the pore or may allow more stable interactions of the structured regions with other cytoplasmic domains and obstruct the pore.

Additional NMR studies have shown that phosphorylation (in response to *src* or other kinases) causes structural changes of the C-terminal domain that may contribute to channel closure or may alter interactions with cellular proteins ^83^. NMR data have also demonstrated structural differences in the CT domains of different connexins ^84^. This result was predictable because the amino acid sequence of the CT domain is highly divergent among members of the connexin family. It had previously been proposed that the extensive differences in the CT sequences of different connexin isoforms likely contribute to differences in channel regulation. The roles played by the structured regions of the CT domain in differential regulation of connexin channels remain to be elucidated.

## Summary and unresolved issues

The different Cx26 crystal structures and the models derived from simulations represent substantial advances for understanding gap junctions. They show many similar features, but they also have some inconsistencies. Currently, simulations produced using the equilibrated Cx26 structure account for experimental data more closely than do the crystal structures. It is possible that ions and larger molecules go through the pore as predicted by the molecular dynamics simulations and that several factors (including charge and/or post-translational modification of the side chains) contribute to determining selective ion/molecular permeability (as explored by Luo *et al*. ^46^).

It is still unclear what determines Ca ^2+^ gating: structural/conformational changes or electrostatic barriers (or both). It is uncertain whether the mechanism involved is similar in hemichannels and in intercellular channels since docking may induce alterations in the positions and involvement of some of the amino acids. Different studies have implicated several amino acids and their interactions in Ca ^2+^-dependent regulation of channel activity, suggesting that this is a complex process. A conformational change of the ELs induced by interaction of Ca ^2+^ with D50 may be the initial step in the closure of Cx26 hemichannels ^75^. Further investigations will be required to resolve the discrepancies and to understand fully all the steps involved.

A future goal will be to develop an accurate representation of the gap junction channel (and hemichannel) in different states (fully open and conducting versus functional substates versus different closed states) under normal conditions and after being gated open or closed by different agents/factors. These studies may clarify whether different gating agents lead to similar or differing conformational changes.

It is uncertain which features of the Cx26 structure will be generalizable to all of the other connexins. Since 20 members of the human connexin family form channels with distinct properties and are differentially expressed in various organs, it will be important to elucidate the channel structures of additional members of the connexin family. Previous studies have suggested that there are critical differences among connexin channels from different subfamilies (e.g. Cx32 versus Cx43 versus Cx36). Biochemical studies have shown dissimilarities among subfamilies, even in conserved regions like the ELs, where the positions of the cysteines forming disulfide bonds may differ ^49, 85^. Since the connexins differ in their primary sequences, the pore-lining amino acids and the charges (and modifications) of their side chains are likely to differ; these differences can influence the characteristics of the pore, resulting in different channel properties (including charge selectivity, size permeability, and single channel conductance). Mathematical modeling suggests that pairing of hemichannels made of different connexins with differences in their pore shapes might account for asymmetrical flux of large charged molecules through heterotypic gap junction channels ^86^. Understanding the structures of different connexin channels and determinants of their specific properties could have valuable therapeutic benefits, such as facilitating the design of inhibitors targeting a particular connexin.
